# LIM homeobox 1 (LHX1) induces endoplasmic reticulum stress and promotes preterm birth

**DOI:** 10.1016/j.heliyon.2024.e32457

**Published:** 2024-06-18

**Authors:** Liyin Qiu, Zhaozhen Liu, Shouzhen Chen, Yiting Wu, Jianying Yan

**Affiliations:** a Department of Obstetrics, Fujian Maternity and Child Health Hospital College of Clinical Medicine for Obstetrics & Gynecology and Pediatrics, Fujian Medical University, Fuzhou, Fujian, 350001, China; b Department of Histology and Embryology, Fujian Maternity and Child Health Hospital College of Clinical Medicine for Obstetrics & Gynecology and Pediatrics, Fujian Medical University, Fuzhou, Fujian, 350001, China

**Keywords:** Preterm birth, LHX1, IRE-1, Autophagy, Endoplasmic reticulum stress, Placental trophoblast cells

## Abstract

**Background:**

Premature birth (PTB) is a major cause of neonatal mortality and has enduring consequences. LIM Homeobox 1 (LHX1) is vital in embryonic organogenesis, while Inositol-Requiring Enzyme 1 (IRE-1) regulates endoplasmic reticulum stress (ERS). This study explores whether IRE-1 impacts PTB via LHX1 modulation.

**Methods:**

We analyzed LHX1 expression in placental samples from PTB patients and examined its impact on the viability, migration, invasion, and apoptosis of the human placental trophoblast cell line HTR8/Svneo, particularly when treated with the ERS inducer tunicamycin (TM). We also assessed the levels of ERS-related genes and autophagy activation in response to LHX1 deficiency. To gain mechanistic insights, we evaluated the ERS-mediated activation of the IRE-1/XBP1/CHOP signaling pathway in LHX1-silenced HTR8/Svneo cells. Additionally, we examined the transcriptional activation of IRE-1 and the binding of LHX1 to the IRE-1 promoter in HTR8/Svneo cells. We overexpressed IRE-1 in LHX1-silenced HTR8/Svneo cells to assess its effects on cell viability, migration, invasion, apoptosis, and autophagy. Finally, we induced LHX1 knockdown in mice through intraperitoneal injections of tunicamycin (TM) and Sh-LHX1 over a 24-h period to evaluate PTB symptoms.

**Results:**

We observed LHX1 overexpression in placental tissue from PTB cases and TM-induced HTR8/Svneo cells. LHX1 depletion enhanced cell viability, migration, and invasion while reducing autophagy and apoptosis. This reduction in LHX1 led to decreased levels of IRE-1, XBP1, CHOP, and other ERS-related genes, indicating LHX1's role in ERS induction and the activation of the IRE-1/XBP1/CHOP pathway. Mechanistically, LHX1 was found to bind to the IRE-1 promoter, inducing its transcriptional activation. Notably, overexpressing IRE-1 counteracted the impact of LHX1 depletion on trophoblast cell behavior, suggesting that LHX1 modulates IRE-1. In line with our in vitro studies, LHX1 knockdown ameliorated PTB symptoms in TM-treated mice.

**Conclusion:**

LHX1 contributes to the progression of PTB by regulating the IRE-1-XBP1-CHOP pathway.

## 1 Introduction

Premature birth (PTB), defined as the delivery of a baby before reaching 37 weeks of gestational age, is a significant global concern [1,2]. Recent data indicates that approximately 13.4 million newborns were born prematurely, and this number continues to rise, emphasizing the urgency of addressing this issue [3]. Infants born prematurely face an increased risk of developing a range of problems, including inflammatory diseases, neurodevelopmental disorders, metabolic issues, and the potential for lifelong disabilities [4,5]. Comprehending the complex pathogenesis of PTB is fundamental for devising innovative treatments and effective preventive measures that will ultimately enhance the well-being of newborns.

During pregnancy, the uterus experiences a series of physiological and biochemical alterations that can lead to the occurrence of endoplasmic reticulum stress (ERS). It has been observed that ERS within the placenta is associated with adverse pregnancy outcomes in cases of complicated pregnancies [[6], [7], [8]]. ERS triggers the unfolded protein response (UPR), a cellular mechanism designed to restore equilibrium within the ER [9,10]. Research indicates that ERS leads to increased uterine caspase-3 and caspase-8 levels at the onset of PTB, potentially affecting fertility [11,12]. Understanding how ERS is involved in PTB may shed light on potential interventions to mitigate its impact, making it a valuable area of study.

LIM Homeobox 1 (LHX1) belongs to the LIM-HD transcription factor family, recognized for its pivotal role in regulating organogenesis during embryonic development [[13], [14], [15]]. LHX1 is a key regulator in endometrial development and remodeling, with the ability to facilitate cell growth and the epithelial-mesenchymal transition (EMT) process in uterine corpus endometrial carcinoma [16]. However, despite these recognized roles, the specific connection between LHX1 and PTB, particularly in the context of ERS, remains largely unexplored. The decision to investigate LHX1 is driven by its regulatory influence on crucial cellular processes in the uterus, prompting the need to unravel its potential contributions to PTB and its association with ERS. Moreover, the intriguing observation that LHX1 is subject to downregulation via GRP78, a vital regulator of ERS and a major sensor for activating the UPR [17], adds complexity to LHX1's potential roles. This intricate relationship positions LHX1 as a compelling focus for research, suggesting potential interplay between LHX1, ERS, and PTB. Thus, our study aims to bridge this knowledge gap by investigating into the uncharted territories of LHX1's involvement in PTB and its connection with the UPR.

Inositol-Requiring Enzyme 1 (IRE-1) is an endoplasmic reticulum (ER) transmembrane protein with the ability to monitor ER luminal stress through its stress-sensing domain, subsequently initiating the UPR via its cytoplasmic kinase domain and RNase domain [18]. Intriguingly, our recent, as-yet-unpublished research highlights that inhibiting the ERS-associated IRE-1 Pathway holds promise in alleviating PTB, indicating a potential connection between IRE-1 and PTB. Nonetheless, it remains uncertain whether LHX1 interacts with IRE-1 and actively participates in the UPR and the progression of PTB. A comprehensive exploration of these aspects is crucial to fully comprehend LHX1's role in PTB and ERS.

In the course of this study, we have identified that LHX1 binds to the IRE-1 promoter, subsequently promoting the transcriptional activation of IRE-1, thus contributing to PTB. These findings offer promising insights that may spark innovative ideas for the diagnosis and treatment of PTB.

## 2 Material and methods

### 2.1 Tissue samples

Placenta samples were collected from a cohort of 30 parturients who experienced preterm births (PTB) and 30 parturients with normal-term pregnancies after the verbal consent. This sampling was conducted at the Fujian Maternity and Child Health Hospital, College of Clinical Medicine for Obstetrics & Gynecology, and Pediatrics, Fujian Medical University. Placental samples were acquired immediately following neonatal delivery and the rupture of placental membranes. Subsequently, these samples were promptly stored at a temperature of −80 °C within a freezer. This study had received prior approval from the Ethics Committee of the Fujian Maternity and Child Health Hospital, College of Clinical Medicine for Obstetrics & Gynecology, and Pediatrics, Fujian Medical University (EA#8896GHW). Furthermore, each participant provided their informed consent to be part of the study.

### 2.2 Cell culture and treatment

The human placental trophoblast cell line, HTR8/SVneo, was procured from Procell (Wuhan, China). These cells were cultured in RPMI 1640 medium (Gibco, #11875093, NY, USA) supplemented with 10 % fetal bovine serum (FBS) and incubated at 37 °C in incubator with 5 % CO_2_. To simulate an ERS environment, the cells HTR8/Svneo were grown aseptically until they reached 80 % confluency and then treated with 5 μg/mL of tunicamycin (TM), (Sigma Aldrich # 11089-65-69) for a duration of 24 h.

### 2.3 Cell transfection

The shRNA against LHX1 (sh-LHX1) with sequence of 5′- GATCCGAGGCTAAGTCTTGTGAACTCGAAAGTTCACAAGACTTAGCCTC -3′ 3′- GCTCCGATCTCGATTCAAGACTTGAATTCTCGAAGTGTTCTGAATCGGAG -5′and the negative control (sh-NC) 5′- GATCCGGAACAGGAGGCGCAACGAATCTCGAAAGATTCGTTGCGCCTCCTGTTCC -3′ 3′- GCTTGTCC TCTTCCGCGTTGCTTAGAAGCTTTCTAAGCAAGCGGAGGAACAA GG -5′ were purchased from Ribobio (Guangzhou, China). The full-length sequence of IRE-1: 5′-ATGCTAGCTAGCTAGCTAGCTAGCTAGCTAGCTAGC-3′) was inserted into pcDNA3.1 vector (Geenseed Biotech, Guangzhou, China) and the empty vector (5′- GTACGACTACGACTACGACTACGACTACGACTACGT-3′) served as NC. Cells were seeded in 6-well plates and grew to 80 % confluence. Lipofectamine 3000 (Invitrogen, L3000001, USA) was utilized for performing cell transfection.

### 2.4 RT-qPCR analysis

Total RNA was extracted using TRIzol (15596026). Subsequently, the RNA underwent reverse transcription for cDNA synthesis, utilizing the Reverse Transcription Kit from Qiagen (Hilden, Germany). Following this, quantitative polymerase chain reaction (qPCR) was performed with the SYBR Green PCR Kit by Takara (Japan) on the StepOnePlus Real-Time PCR System by Applied Biosystems (USA). To quantify RNA expression levels, the 2^−ΔΔCt^ method was applied, with GAPDH serving as the control.

The primer sequences are presented in Table 1.

**Table 1:** Primers used in RT-qPCR.

Gene	Forward sequence	Reverse sequence
**XBP-1**	5′-CTGTGAGCGAGTCCGAATC-3′	5′-AGTCAATACCGCCAGAATGC-3′
**LHX-1**	5′- ATGGAGTACCTGAGGTGGCG -3′	5′- CGCCACTCAGGTACTCCAT -3′
**GRP78**	5′-GCTTGTGGGTCTGGAGAAGA-3′	5′-GACATCTTCTCCTGCGTCTG-3′
**CHOP**	5′-ACAGAGGAGGAGGAGGAGGA-3′	5′-CTGTTCTCCTTCTCCTGCTG-3′
**EIF2-α**	5′-GCTGTTGGTGAAGGAGGAGA-3′	5′-TGTCCAGTTCTGCTCCCTTC-3′
**IRE-1**	5′-TGCTTGGTGATGTGGAAAGG-3′	5′-AGTTCTGGCTGGGTGTCTTT-3′
**ATF6**	5′-AGAGCGCTTCGAGAGGAAGT-3′	5′-GGGTTTCTGTCCAGCTCCTT-3′

### 2.5 Immunofluorescence (IF)

HTR8/SVneo cells were seeded on glass slides in 24-well plates. After fixing them with 4 % formaldehyde (Sigma# 30525-89-4), the cells were treated with 0.1 % Triton X-100 (Sigma Aldrich# 9036-19-5). Subsequently, the cells were incubated with *anti*-LC3B (Abcam, ab#48394) at a dilution of 1:500 or *anti*-LHX1 (Abcam, #ab229474) at a dilution of 1:900 at 4 °C overnight. Following this, the cells were washed and incubated with a goat anti-rabbit secondary antibody (Abcam, #ab97075) for 2 h. DAPI (ThermoFisher Scientific #62248) was used to stain the cell nuclei and cells were finally observed using an Olympus microscope (Tokyo, Japan).

### 2.6 Cell viability assays

Cells (2 × 10^3^) were seeded in 96-well plates and allowed to incubate for 24 h. Subsequently, they were treated with 10 μl of CCK-8 (Sigma Aldrich #96992) at various time points for an additional 2 h. The absorbance at 450 nm was measured using a microplate reader (Tecan Infinite, Mannedorf, Switzerland).

### 2.7 Colony formation assays

Colony formation assays were conducted by seeding approximately 1000 cells into a 60-mm dish. Following a 14-day incubation in a uniform atmosphere with 5 % CO2 at 37 °C, the resultant clones were fixed using methyl alcohol and stained with crystal violet (C0121, Beyotime, Shanghai, China) for 30 min. Afterward, they were washed with ddH2O and manually counted under a microscope.

### 2.8 Transwell assay

Migratory and invasive capabilities of HTR8/SVneo cells were evaluated using Transwell chambers (8 μm pore size, Corning #3428) with or without Matrigel (BD Biosciences, USA #356234). Cells were seeded in the upper chamber of the Transwell with serum-free RPMI medium, while the lower chamber was filled with complete culture medium. After 24 h, cells that had migrated to the lower chamber were fixed with 4 % paraformaldehyde (Sigma #30525-89-4), stained with crystal violet (Sigma #548-62-9), and observed under a microscope (Olympus).

### 2.9 Flow cytometry

Cell apoptosis in HTR8/SVneo cells was assessed using the Annexin V/Dead Cell Apoptosis kit (Thermo Fisher Scientific, USA, #V13242). After transfection, cells were double-stained with 5 μl of Annexin V-FITC and 5 μl of propidium iodide (Thermo Fisher, #BMS500PI) in the dark for 15 min. Subsequently, a FACS Calibur flow cytometer (BD Biosciences, USA) in conjunction with FlowJo software was employed to measure the cell apoptosis rate.

### 2.10 Western blot

Total proteins were extracted using RIPA buffer (Sigma Aldrich#R0278). After electrophoresis on SDS-PAGE, the proteins were transferred onto PVDF membranes and blocked with 5 % nonfat milk. Subsequently, they were incubated with primary antibodies, including LHX-1 (ab#229474, 1:100), GAPDH (ab#9485, 1:1000), LC3 I & II (Millipore Sigma#ABC929, 1:300), p62 (Cell Signaling#5114, 1:600), ATG5 (Cell Signaling#2630, 1:450), Beclin 1 (Cell Signaling#3738, 1:700), IRE-1 (Abcam ab#48187, 1:1500), CHOP (Proteintech#15204-1-AP, 1:700), ATF6 (Abcam#ab227830, 1:300), GRP78 (Abcam ab#21685, 1:900), EIF2-α (Santa Cruz#sc133132, 1:500), and p-EIF2-α (CST#9721, 1:600), at 4 °C overnight. This was followed by incubation with secondary antibodies (Invitrogen, Goat anti-rabbit Cat # 31460–1:400, and goat anti-mouse, 31430) for 2 h. Protein bands were detected using the ECL kit (Millipore, Billerica, MA, USA) and analyzed using ImageJ.

### 2.11 ChIP assay

Cells were initially cross-linked using 1 % formaldehyde. Subsequently, lysis buffer was introduced to the treated cells, and the chromatin was fragmented into DNA fragments of 150–900 bp through sonication. Following this, *anti*-LHX1 or a control *anti*-IgG was included in the sonicated mixtures for incubation. The precipitated complexes were then subjected to washing and reverse cross-linking. After purification, the isolated DNA was analyzed using RT-qPCR.

### 2.12 Luciferase reporter assay

Luciferase reporter assay was carried out to assess the impact of LHX1 binding on the IRE-1 promoter's regulatory activity. Briefly, the wild-type (WT) or mutated (Mut) LHX1 binding sites within the IRE-1 promoter region were sub-cloned into the luciferase reporter vector pGL3 (Promega, Madison, WI). Subsequently, cells were co-transfected with the reporter vectors and the specified plasmids. After a 48-h incubation period, the Luciferase Reporter Assay System (Promega) was utilized to conduct the luciferase activity analysis.

### 2.13 PTB mouse model

We induced a PTB mouse model using the ERS inducer, TM. Nakashima et al. showed that intraperitoneal injection of TM successfully initiated a premature increase in myometrial gap junction protein alpha 1 levels, elevated contractile responsiveness, and advanced the onset of preterm labor in mice. These effects were attributed to the induction of an exaggerated UPR [19]. Prior to conducting this experiment, we obtained the necessary ethical clearance from the Ethics Committee of the Fujian Maternity and Child Health Hospital College of Clinical Medicine for Obstetrics & Gynecology and Pediatrics, Fujian Medical University (EA#A227891). For this study, timed pregnant CD1 mice were procured from the Fujian Maternity and Child Health Hospital College of Clinical Medicine for Obstetrics & Gynecology and Pediatrics, Fujian Medical University, and they were raised in a controlled SPF environment. On E13, pregnant mice underwent one of the following treatments: a vehicle injection (sh-NC, 5′-GACGTTCAGGCTTGCACTTGA-3′), an intraperitoneal injection of 0.2 mg/kg TM, or a combined injection of TM + Sh-LHX1, with the objective of delivering small hairpin RNA (shRNA) 5'-(GCTGCAAGGCTGACTTACGTT-3′) targeting the LHX1 gene. These treatments were administered over a 24-h period. Mice in the TM + Sh-LHX1 group received an intravenous injection of 500 μg of shRNA treatment in 0.5 mL of PBS via the tail vein 1 h after the administration of TM. Subsequently, the mice were euthanized, or they were monitored until they reached full term. Placenta samples were collected, and they were promptly stored at −80 °C for subsequent analysis.

### 2.14 Immunohistochemistry (IHC)

Placenta samples were sectioned into 5-μm slices, and the slides were passed through three changes of xylene followed by graded ethanol for deparaffinization and rehydration. Antigen retrieval from the tissues was performed by heating them in a sodium citrate buffer (Sigma, #C2488) in an oven. Subsequently, the slides were treated with 3 % hydrogen peroxide (Sigma#7722-84-1) for 15 min and then blocked with goat serum. The tissue sections were then incubated with primary antibodies against LHX1 (ab#229474, 1:250) and LC3 (Millipore Sigma#ABC929, 1:100) overnight at 4 °C. Following this, they were incubated with a goat anti-rabbit secondary antibody (Invitrogen, Goat anti-rabbit Cat # 31460, 1:400) for 2 h. After thorough washing with phosphate buffer saline, the slides were treated with DAB substrate (Abcam#, ab64238) for 10 min and counterstained with hematoxylin (Sigma#517282). Finally, the slides were washed again and mounted with mounting media (Enzo life sciences # ADI-950261) before being observed using a microscope (Olympus, Japan).

### 2.15 TUNEL assay

The tissue slides underwent dewaxing with xylene (Sigma#1330207, St. Louis, MO, USA), rehydration through varying ethanol concentrations (Sigma#64175, St. Louis, MO, USA), and a subsequent permeation in 0.1 % Triton X-100 in PBS for 5 min. Following this, Equilibration Buffer (100 μl) was applied to the slides at room temperature for 10 min. Staining was executed using the TUNEL assay kit, In Situ Cell Death Detection Kit (Roche, Basel, Switzerland), as per the provided instructions. Apoptotic cells were observed at a magnification of × 100 using an Olympus microscope (Tokyo, Japan). To quantify apoptotic cells, eight randomly chosen fields were selected for manual counting, and image analysis was conducted using Motic Med 6.0 software (Xiamen Motic Software Engineering Co., Ltd., Xiamen, China). The apoptosis index was then determined by dividing the number of apoptotic cells by the total number of cells and multiplying by 100 %.

### 2.16 Statistical analyses

The data were reported as means ± standard deviation (SD) derived from three independent replicates. Statistical analysis was conducted using GraphPad Prism 6 software, employing either a Student's t-test or a one-way analysis of variance (ANOVA). A significance threshold of P < 0.05 was applied to ascertain statistical significance.

## 3 Results

### 3.1 LHX1 expresses at a high level in PTB placenta

**Fig. 1:**
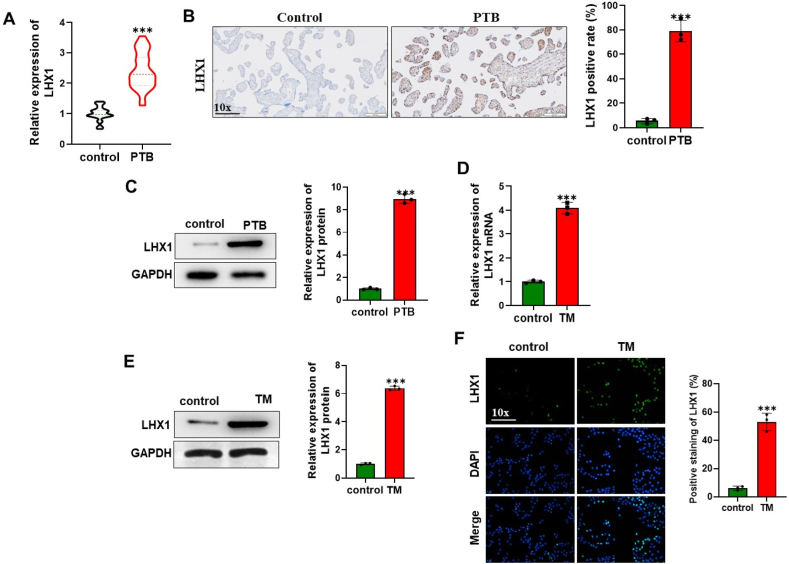
Elevated LHX1 Expression in PTB Placenta. *(A) RT-qPCR analysis comparing LHX1 expression in normal term placenta (n* = *30) and PTB placenta (n* = *30), illustrating a substantial upregulation of LHX1 in PTB samples. (B) IHC staining highlights increased LHX1 expression in the representative sections of the placenta of control group (n* = *3) and PTB group (n* = *3) providing visual confirmation of heightened LHX1 levels in PTB tissues. (C) Western blot results indicating elevated LHX1 protein levels in the placenta of control group (n* = *3) and PTB group (n* = *3). (D-E) RT-qPCR and Western blot analysis of LHX1 expression in control and TM-treated HTR8/Svneo cells (n* = *3 per group). Both mRNA and protein levels of LHX1 exhibit marked increases upon TM induction, indicating ERS-induced upregulation of LHX1. (F) IF assay demonstrates increased LHX1 expression in control and TM-treated HTR8/Svneo cells (n* = 3 per group), further supporting the observed increase in LHX1 expression. ***P < *0.001 vs control.*

In our initial investigation, we assessed LHX1 expression in placentas affected by PTB. The results from RT-qPCR revealed a significant upregulation of LHX1 expression in PTB placenta compared to normal full-term placenta (Fig. 1A). Furthermore, IHC analysis corroborated this heightened expression of LHX1 in PTB placenta (Fig. 1B). Western blot analysis provided additional confirmation of elevated LHX1 protein levels in PTB placenta (Fig. 1C). Subsequently, we established a cellular model of ERS injury in PTB by treating human trophoblast cells with tunicamycin (TM) and examined LHX1 expression in these cells. Both LHX1 mRNA and protein levels exhibited marked increases upon TM induction (Fig. 1D and E). Additionally, IF results demonstrated strong fluorescence intensity of LHX1 in TM-induced cells (Fig. 1F). Overall, our results demonstrated that increased expression of LHX1 in PTB might play a role in regulating PTB development.

### 3.2 LHX1 promotes autophagy and apoptosis of trophoblast cells

We next treated HTR8/Svneo cells with TM, and the impact of TM on cellular behaviors and ERS-related markers was evaluated. Our findings demonstrate that TM exerted significant effects on HTR8/Svneo cells, leading to the suppression of cell viability, proliferation, migration, and invasion. Moreover, TM treatment resulted in the promotion of autophagy in HTR8/Svneo cells, accompanied by an induction of ERS markers, including XBP-1, CHOP, AF6, and GRP78 (Fig. S1).

**Fig. 2:**
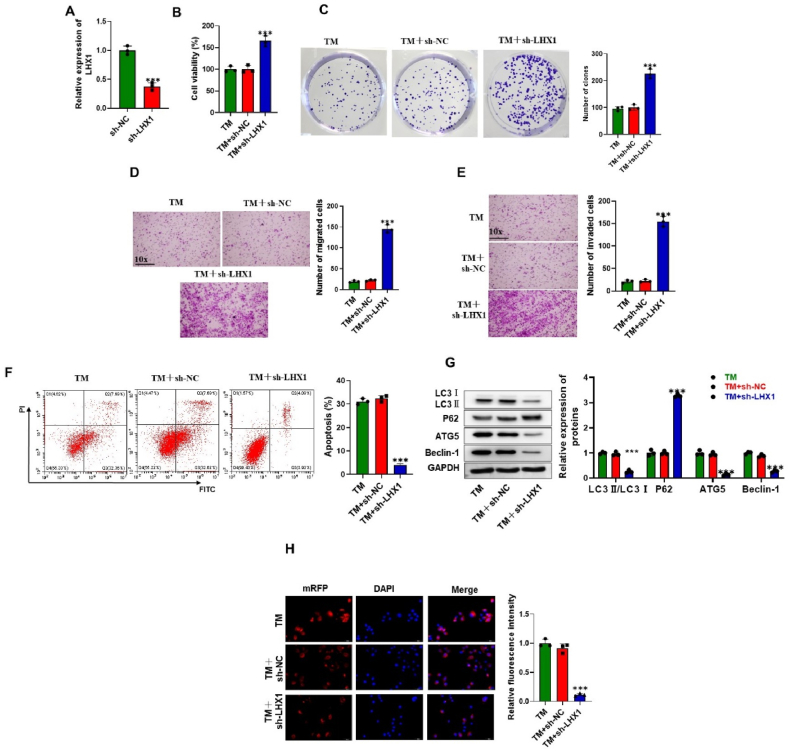
**LHX1 promotes autophagy and apoptosis of trophoblast cells.** (A) RT-qPCR results demonstrating the transfection efficiency of sh-LHX1 in HTR8/Svneo cells induced with TM (n = 3). (B–C) Cell viability and proliferation of HTR8/Svneo cells were assessed by CCK-8 and colony formation assays after treatment with TM and transfection with sh-NC or sh-LHX1 (n = 3 per group). (D–E) Evaluation of cell migratory and invasive capabilities using transwell assays (n = 3). Flow cytometry analysis was performed to determine the cell apoptotic rate after treatment with TM and transfection with sh-NC or sh-LHX1 (n = 3 per group). (G) Western blot results depicting protein levels of LC3, P62, ATG5, and Beclin-1 in HTR8/Svneo cells after treatment with TM and transfection with sh-NC or sh-LHX1 (n = 3 per group). (H) Immunofluorescence (IF) assay for assessing LC3 1 in HTR8/Svneo cells after treatment with TM and transfection with sh-NC or sh-LHX1 (n = 3 per group). ***P < 0.001 vs sh-NC.

We then knocked down LHX1 expression in cells to investigate its impact on cellular behavior. RT-qPCR results showed the transfection efficiency of sh-LHX1 in HTR8/Svneo cells induced with TM (Fig. 2A). Interestingly, the knockdown of LHX1 significantly increased the viability and proliferation of cells treated with TM compared to cells induced with TM but transfected with sh-NC (Fig. 2B and C). Transwell assays confirmed that silencing LHX1 enhanced cell migratory and invasive capabilities under TM stimulation (Fig. 2D and E). Subsequently, we observed a significant decrease in the rate of cell apoptosis in the TM + sh-LHX1 group (Fig. 2F). We next examined the impact of LHX1 silencing on autophagy by monitoring levels of autophagy markers. Our results indicated a reduction in the ratio of LC3II/LC3I, decreased levels of ATG5 and Beclin-1, and increased levels of P62 in cells induced with TM due to LHX1 silencing, suggesting the inhibition of autophagy (Fig. 2G). These results were further confirmed by the diminished fluorescence intensity of the autophagy marker LC3 in TM + sh-LHX1-induced cells (Fig. 2H). Therefore, our findings suggest that under TM stimulation, LHX1 restrains the migratory and invasive capabilities of trophoblast cells, while promoting autophagy and apoptosis however all of these effects were reversed in absence of LHX1.

### 3.3 LHX1 regulates the IRE-1/XBP1/CHOP signaling pathway

**Fig. 3:**
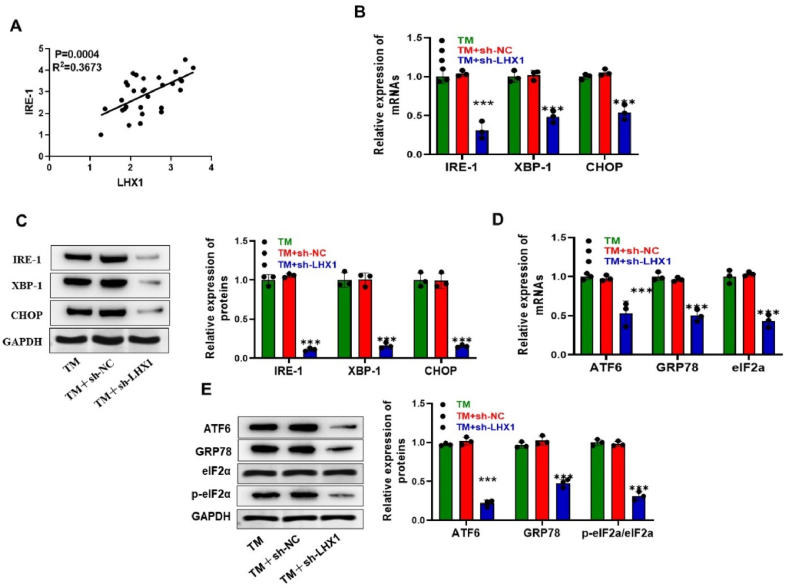
**LHX1 modulates the IRE-1/XBP1/CHOP signaling pathway**. (A) Assessment of the expression correlation between IRE-1 and LHX1 in 30 preterm birth (PTB) placenta samples. The analysis, performed using Pearson's correlation, indicates a significant correlation (P = 0.0004, R2 = 0.3673), suggesting a potential functional association between IRE-1 and LHX1. (B–C) RT-qPCR and Western blot analysis results showing the mRNA and protein expression levels of IRE-1, XBP-1, and CHOP in HTR8/Svneo cells from the TM group, TM + sh-NC group, and TM + sh-LHX1 group (n = 3 per group). (D–E) RT-qPCR and Western blot analysis results illustrating the mRNA and protein expression levels of ATF6, GRP78, and eIF2α in HTR8/Svneo cells of TM group, TM + sh-NC, and TM + sh-LHX1 groups (n = 3 per group). ***P < 0.001 vs TM + sh-NC.

IRE is the ER transmembrane receptor and IRE-1/XBP1/CHOP signaling pathway plays a crucial role in maintaining ER stability and is a key component in ERS. This prompted us to investigate the relationship between IRE-1 and LHX1. Interestingly, Pearson's correlation analysis indicated a positive regulatory connection between LHX1 and IRE-1 (Fig. 3A). This led us to hypothesize that LHX1 might be involved in the development of preterm birth (PTB) by influencing the IRE-1/XBP1/CHOP pathway.

To test this hypothesis, we conducted experiments to observe mRNA and protein levels of IRE-1, XBP1, and CHOP and ERS related genes. Our results revealed a significant decrease in IRE-1, XBP1, and CHOP mRNA and protein levels when LHX1 was knocked down in TM-induced HTR8/Svneo cells (Fig. 3B and C). Furthermore, we observed that the deficiency of LHX1 led to a reduction in the mRNA levels of ERS-related genes ATF6, GRP78, and eIF2α (Fig. 3D). Western blot analysis confirmed a notable decrease in ATF6, GRP78, and p-eIF2α levels in response to LHX1 deficiency (Fig. 3E). These findings provide strong evidence that LHX1 activates the IRE-1/XBP1/CHOP signaling pathway, thus contributing to PTB development.

### 3.4 Transcriptional activation of IRE-1 expression by LHX1

**Fig. 4:**
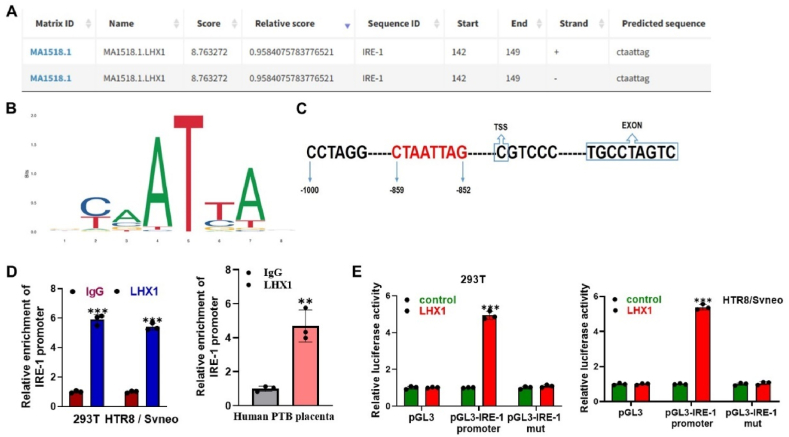
**LHX1 transcriptionally activates IRE-1 expression.** (A) Predicted binding sites of LHX1 on the IRE-1 promoter obtained from the JASPAR database, laying the foundation for investigating the physical interaction between LHX1 and IRE-1. (B) DNA motif of LHX1 obtained from the JASPAR database, providing a sequence diagram for a detailed insight into the binding sites. (C) Schematic representation illustrating the interaction between LHX1 and the IRE-1 promoter, highlighting the predicted binding sites. (D) ChIP assay results demonstrating the binding of LHX1 to the IRE-1 promoter in 293T, HTR8/Svneo cells, and human PTB placentas (n = 3 per group). *Anti*-IgG antibody served as the negative control for the *anti*-LHX1 antibody. (E) Luciferase reporter assays confirming the transcriptional activation effect of LHX1 on the IRE-1 Promoter in 293T and HTR8/Svneo cells (n = 3 per group). Cells were co-transfected with pcDNA3.1 vector expressing LHX1 and pGL3 vector expressing wild type or mutated IRE-1 promoter. **P < 0.01, ***P < 0.001 vs IgG or control.

We next aimed to investigate the physical interaction between LHX1 and IRE-1. We thus utilized the JASPAR database to predict the binding sites of LHX1 and IRE-1 (Fig. 4A). To provide a more detailed insight, we further depicted the sequence diagram of the LHX1 motif (Fig. 4B) and the binding sites between LHX1 and the IRE-1 promoter (Fig. 4C). To experimentally validate this interaction, we conducted a ChIP assay, and the results clearly demonstrate that the enrichment of the IRE-1 promoter was significantly elevated in the LHX1 group when compared to the IgG control in HTR8/Svneo cells and human PTB placentas (Fig. 4D). Moreover, we assessed the functional consequences of LHX1 binding to the IRE-1 promoter. Notably, the luciferase activity of the wild-type IRE-1 promoter was significantly enhanced in response to LHX1 overexpression, whereas this effect was not observed with IRE-1 mutants, as demonstrated in both 293 T and HTR8/Svneo cells (Fig. 4E). Taken together, these findings provide strong evidence that LHX1 specifically targets the IRE-1 promoter and exerts a transcriptional activating effect on its expression.

### 3.5 LHX1 regulates trophoblast cell behaviors by upregulating IRE-1

**Fig. 5:**
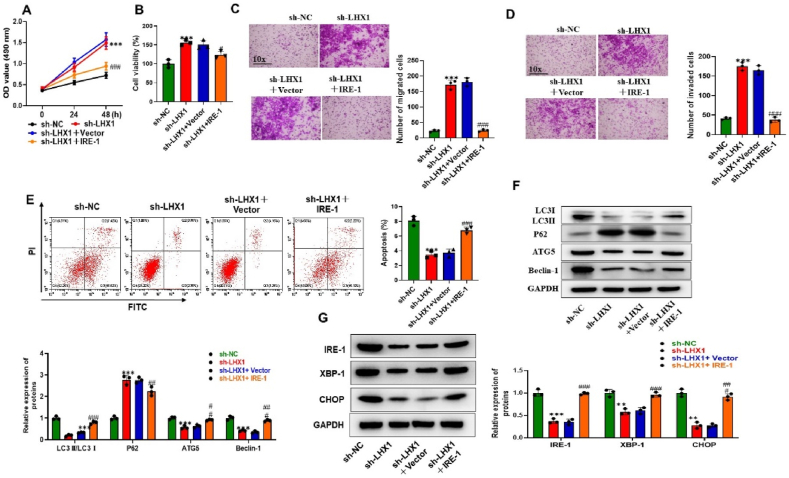
LHX1 Upregulation Modulates Trophoblast Cell Behaviors Through IRE-1. (A–B) CCK-8 and MTT assays were employed to evaluate cell viability in TM-induced HTR8/Svneo cells across various groups, including the sh-NC group, the sh-LHX1 group, the sh-LHX1+Vector group, and the sh-LHX1+IRE-1 group (n = 3 per group). (C–D) Cell migratory and invasive capabilities of sh-NC group, sh-LHX1 group, sh-LHX1+Vector group, and sh-LHX1+IRE-1 groups were assessed through Transwell assays (n = 3 per group). (E) Cell apoptotic rates of sh-NC group, sh-LHX1 group, sh-LHX1+Vector group, and sh-LHX1+IRE-1 groups were determined by flow cytometry analysis (n = 3 per group). (F–H) Western blot analysis was performed to examine the protein levels of LC3, P62, ATG5, Beclin-1, IRE-1, XBP-1, and CHOP in HTR8/Svneo cells of sh-NC group, sh-LHX1 group, sh-LHX1+Vector group, and sh-LHX1+IRE-1 groups (n = 3 per group). **P < 0.01, ***P < 0.001 compared with the sh-NC group; #P < 0.05, ##P < 0.01, ###P < 0.001 compared with the sh-LHX1+Vector group.

Our next goal was to examine how LHX1, and IRE-1 impact the regulation of trophoblast cell functions. We have shown in previous results that silencing LHX1 led to an enhancement in cell proliferation and viability. However, these effects were significantly reversed when IRE-1 was overexpressed (Fig. 5A and B). Furthermore, transwell assay revealed that IRE-1 overexpression effectively countered the migratory and invasive capabilities caused by LHX1 knockdown in TM-induced HTR8/Svneo cells (Fig. 5C and D). Moreover, when assessing the apoptotic rate of cells, we observed that inhibitory effect on apoptosis due to LHX1 deficiency was rescued or even enhanced when IRE-1 was overexpressed (Fig. 5E). Additionally, our study examined that IRE-1 upregulation reversed the inhibitory effect of LHX1 deficiency on protein levels of LC3II/LC3I, ATG5 level, Beclin-1 level, and reversed the promoting effect on P62 level (Fig. 5F). Moreover, we delved into the activity of the IRE-1/XBP1/CHOP pathway, which was found to be suppressed by LHX1 silencing as shown in previous results. However, when IRE-1 was upregulated, it effectively reactivated this pathway (Fig. 5G). In summary, our results collectively highlight that LHX1 plays a crucial role in regulating the migration, invasion, apoptosis, and autophagy of trophoblast cells. It accomplishes this by upregulating IRE-1, thereby underscoring the intricate interplay between these two factors in the context of trophoblast cell behavior.

### 3.6 LHX1 knockdown alleviates PTB symptom in mice

**Fig. 6:**
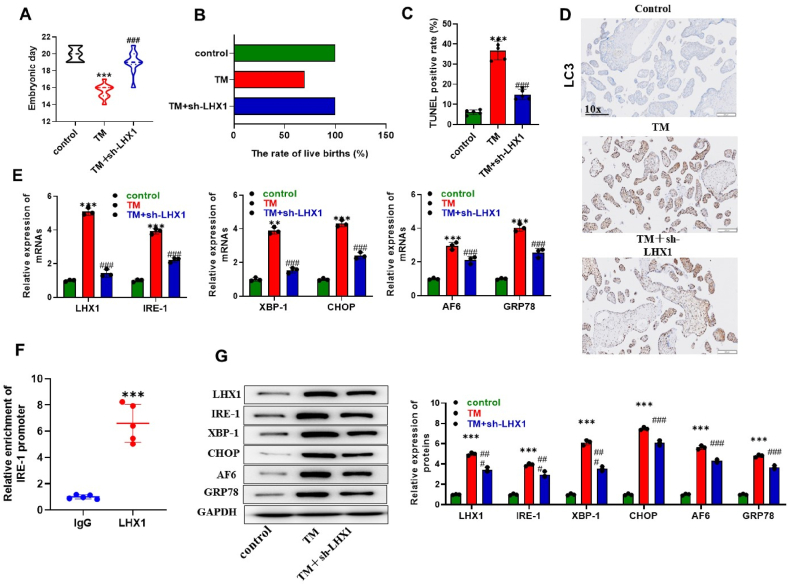
**LHX1 knockdown reduces PTB symptom in mice.** (A) Statistical analysis of the pregnancy duration in mice across three groups: the control group, the TM group, and the TM + sh-LHX1 group (n = 5 per group). (B) Assessment of the live birth rate in control group, TM group, and TM + sh-LHX1 group (n = 5 per group). (C) Measurement of the rate of cell apoptosis in placental tissues of control group, TM group, and TM + sh-LHX1 group using the TUNEL assay (n = 5 per group). (D) Visualization of LC3-positive regions in placental tissues of control group, TM group, and TM + sh-LHX1 group through IHC (n = 3 per group). (E) Evaluation of mRNA levels of LHX1, IRE-1, XBP-1, CHOP, AF6, and GRP78 in placental tissues of control group, TM group, and TM + sh-LHX1 group, conducted via RT-qPCR (n = 3 per group). (F) A ChIP assay was used to assess the binding of LHX1 on IRE-1 promoter in placenta tissues of PTB mice. (G) Western blot was performed to assess protein levels of LHX1, IRE-1, XBP-1, CHOP, AF6, and GRP78 in placental tissues of control group, TM group, and TM + sh-LHX1 group (n = 3 per group). **P < 0.01, ***P < 0.001 compared with the control group; ###P < 0.001 compared with the TM group.

We established a PTB mouse model to confirm the *in vivo* role of LHX1 in PTB development. The mice were divided into three groups (n = 5/group): a control group, a group subjected to TM treatment to induce ERS, and a group treated with both TM and Sh-LHX1 to simultaneously reduce LHX1 gene expression. Mice subjected to TM treatment had a shortened pregnancy period, approximately 16 days, indicative of PTB development. In contrast, mice receiving TM-sh-LHX1 treatment had a normal pregnancy duration, generally lasting 20 days (Fig. 6A). Furthermore, the live birth rate in the TM-treated mice was lower than that of the control group, while the TM-sh-LHX1-treated mice exhibited a higher live birth rate than the TM-treated group (Fig. 6B). To investigate the impact on placental tissue, we conducted TUNEL assays to assess apoptosis. We observed a notable increase in the rate of apoptosis in the TM group, while the TM-sh-LHX1-treated mice exhibited a reduction in the high apoptosis rate induced by TM (Fig. 6C). Additionally, to assess the effects on autophagy, we examined the expression of LC3. IHC confirmed enhanced LC3 expression in the placenta of the TM group. However, LHX1 knockdown led to reduced LC3 expression, indicating the inhibition of autophagy, consistent with our in vitro findings (Fig. 6D). Furthermore, we investigated the expression of LHX1, IRE-1, XBP-1, CHOP, AF6, and GRP78 at both mRNA and protein levels. TM induction resulted in elevated mRNA expression of these factors, while LHX1 knockdown had the opposite effect, aligning with our in vitro results (Fig. 6E). Furthermore, our analysis revealed that LHX1 significantly bound to the IRE-1 promoter in PTB placentas, demonstrating a potential regulatory role (Fig. 6F). Additionally, silence of LHX1 reduced IRE-1, XBP-1, CHOP, AF6, and GRP78 protein levels in placentas of PTB mice (Fig. 6G). These findings collectively suggest that silencing LHX1 can ameliorate the PTB phenotype in mice.

## 4 Discussion

This study investigates the association between ERS, specifically mediated by the UPR, and PTB. In the development of the placenta, invasive extravillous trophoblasts (EVTs) play a critical role by facilitating the production of pregnancy hormones and performing key functions, including fetal immune defense [20]. The inadequacy of EVT invasion can lead to acute obstetrical complications, including PTB [21]. Our results reveal a significant upregulation of LHX1 in PTB placenta, both at the mRNA and protein levels. The induction of ERS in trophoblast cells further corroborated the marked increase in LHX1 expression under the influence of TM, highlighting its potential role in regulating PTB development. In exploring the functional implications of LHX1, our study demonstrates its impact on trophoblast cell behaviors, including migration, invasion, apoptosis, and autophagy, particularly when influenced by TM stimulation. LHX1 appears to exert these effects by modulating the IRE-1/XBP1/CHOP signaling pathway. The intricate relationship between LHX1 and IRE-1 is further supported by our findings that LHX1 transcriptionally activates IRE-1 expression, establishing a novel regulatory axis in the context of PTB. Furthermore, significance of LHX1 extends beyond the trophoblast domain, marking a pivotal presence during the emergence of the anterior visceral endoderm in utero [22]. Our *in vivo* findings align with our in vitro data, demonstrating that silencing LHX1 alleviates PTB symptoms, normalizing pregnancy duration and live birth rates while reducing apoptosis and modulating autophagy in placental tissues. These comprehensive results collectively underscore the indispensable role of LHX1 in placental development and its potential impact on the intricate pathways leading to PTB.

Autophagy represents a highly conserved degradation pathway to maintain cellular equilibrium [19] and plays a pivotal role in embryonic and placental development [19]. Augmented autophagy is associated with the rupture of the amniotic membrane that envelops the fetuses during pregnancy [23]. Variations in placental autophagy features have been observed in PTB [24]. Furthermore, autophagy can be induced by the UPR, and key ER proteins involved in modulating autophagy encompass PERK, IRE-1, and ATF6 [25,26]. LC3 is commonly employed as an autophagosome marker. Other frequently used genes related to autophagy encompass ATG5, Beclin-1, and P62. P62 serves as an autophagy adaptor protein, facilitating the selective degradation of proteins [[27], [28], [29]]. In our study, we have established that the knockdown of LHX1 inhibits cell apoptosis, reduces the levels of LC3, ATG5, and Beclin-1, and increases P62 levels. These findings suggest that LHX1 deficiency suppresses autophagy in TM-stimulated HTR8/SVneo cells.

IRE-1 is an ER luminal stress-sensing domain to monitor conditions and activates the UPR [18]. When the ER experiences an accumulation of unfolded or misfolded proteins, IRE-1 dissociates from GRP78, resulting in the splicing of XBP1 mRNA to produce XBP1s [30]. Notably, XBP1s exhibits the remarkable ability to bind to ERS response elements found in the promoters of numerous UPR target genes. This, in turn, aids in folding and degrading proteins, ultimately promoting ER adaptation and cellular protection [31]. Our investigation revealed a novel facet of IRE-1 function, wherein it transcriptionally activates LHX1 by binding to its promoter specifically in TM-stimulated HTR8/Svneo cells and placental tissues. The significance of IRE-1 in placental development and embryonic viability has been well-established [32]. Intriguingly, experiments involving the overexpression of IRE-1 showcased its capability to counteract the effects of LHX1 depletion. Specifically, it mitigated the impact on cell migration, invasion, apoptosis, and autophagy in HTR8/Svneo cells. These findings provide compelling evidence for the substantial contribution of IRE-1 in ERS-induced PTB, shedding light on its pivotal role in the intricate molecular pathways governing placental dynamics. Notably, under severe and prolonged stress, ERS can trigger the apoptotic pathway [25,31]. This UPR-mediated apoptosis pathway is a novel one, and it involves the activation of CHOP [33], a transcription factor that induces cell apoptosis and is regulated by XBP1 [34,35]. Activation of the IRE-1/XBP1/CHOP pathway has been demonstrated to promote cell apoptosis and inflammatory reactions, leading to the disruption of the intestinal barrier [36]. Additionally, reports indicate elevated expression levels of GRP78, IRE-1, and XBP1 in PTB fetal membranes, which are associated with embryonic lethality and inhibiting ERS may hold promise as a potential therapeutic strategy for addressing PTB [6,37,38]. In our study, LHX1 knockdown significantly reduces the levels of IRE-1, XBP1, and CHOP in TM-induced HTR8/Svneo cells. Additionally, mRNA levels of ERS-related genes ATF6, GRP78, and eIF2α are inhibited by the deficiency of LHX1. Consequently, we can confirm that LHX1 activates the IRE-1/XBP1/CHOP signaling pathway in PTB. In summary, our study provides comprehensive insights into the regulatory roles of LHX1 in PTB, unraveling its connections to ERS and the IRE-1/XBP1/CHOP pathway. These findings open avenues for further research and potential therapeutic interventions aimed at mitigating the impact of PTB.

## Ethics approval and consent to participate

The experimental protocol was established according to the ethical guidelines of the *Helsinki Declaration* and was approved by Ethics Committee of the Fujian Maternity and Child Health Hospital, College of Clinical Medicine for Obstetrics & Gynecology, and Pediatrics, Fujian Medical University (EA#8896GHW). The animal studies received approval from the Ethics Committee of the Fujian Maternity and Child Health Hospital College of Clinical Medicine for Obstetrics & Gynecology and Pediatrics, Fujian Medical University (EA#A227891).

## Funding

This work was supported by the Joint Funds for the innovation of science and Technology, Fujian province (No. 2020Y9142).

## Data availability statement

The data that support the findings of this study are available from the corresponding author upon reasonable request.

## CRediT authorship contribution statement

**Liyin Qiu:** Writing – original draft, Software, Project administration, Methodology. **Zhaozhen Liu:** Writing – review & editing, Methodology, Formal analysis, Data curation. **Shouzhen Chen:** Writing – review & editing, Project administration, Methodology, Data curation. **Yiting Wu:** Writing – review & editing, Software, Formal analysis. **Jianying Yan:** Resources, Investigation, Funding acquisition.

## Declaration of competing interest

The authors declare that they have no known competing financial interests or personal relationships that could have appeared to influence the work reported in this paper.
