# Supplementary figures and images for: LIM homeobox 1 (LHX1) induces endoplasmic reticulum stress and promotes preterm birth
Source: Heliyon. 2024 Jun 18;10(13):e32457. doi: 10.1016/j.heliyon.2024.e32457 (PMC467042; doi:10.1016/j.heliyon.2024.e32457)

Fig 1C


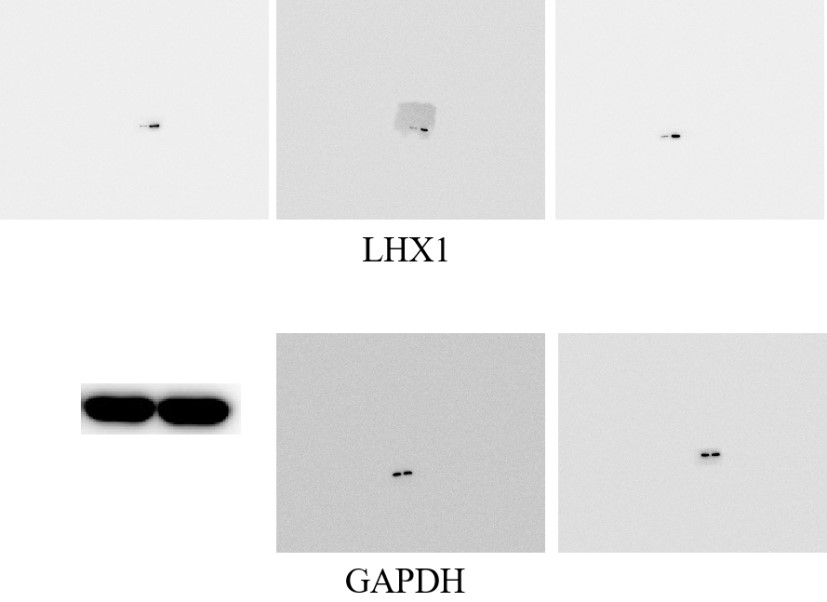


Fig 1E


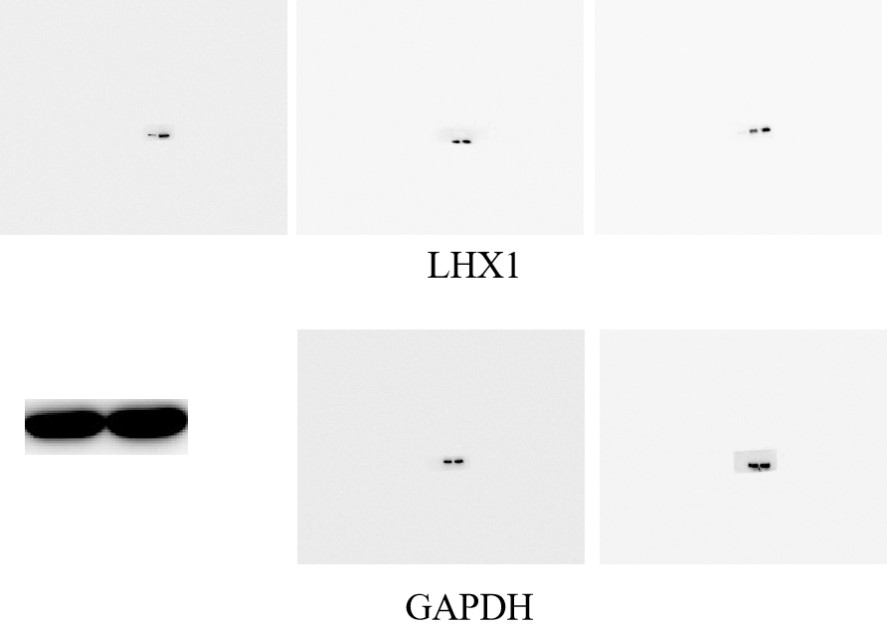


Fig 2G


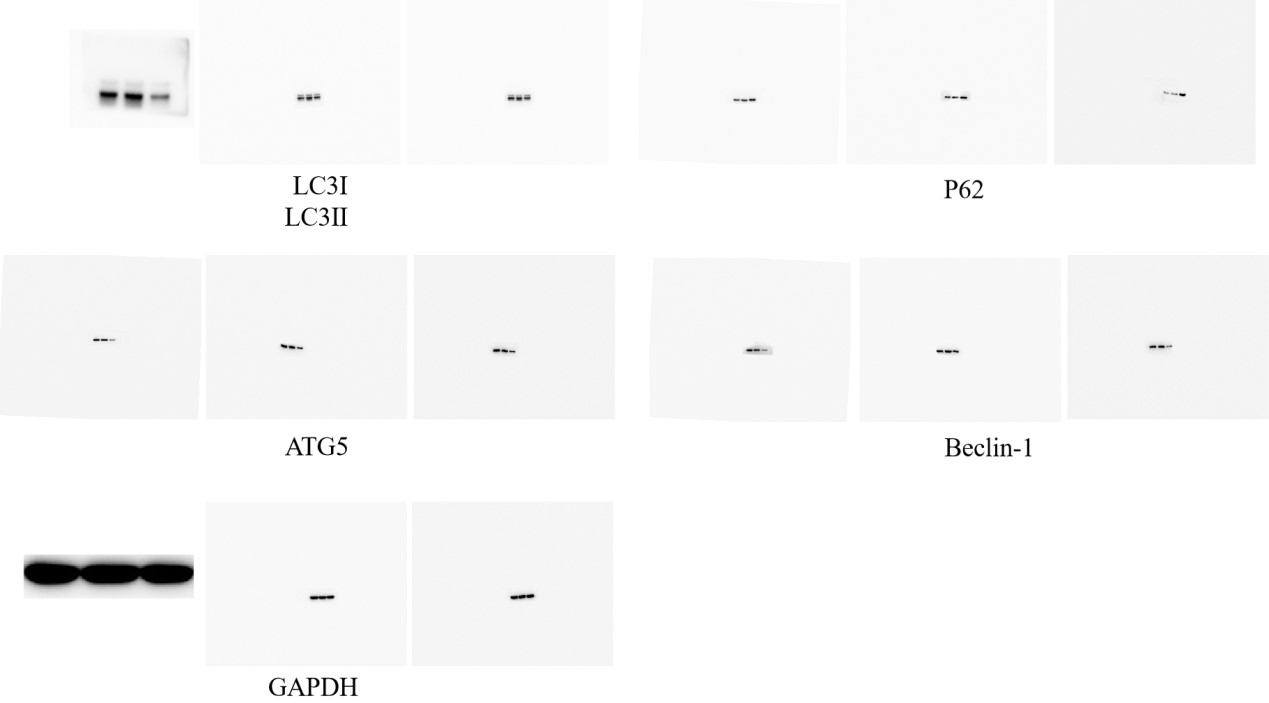


Fig 3C


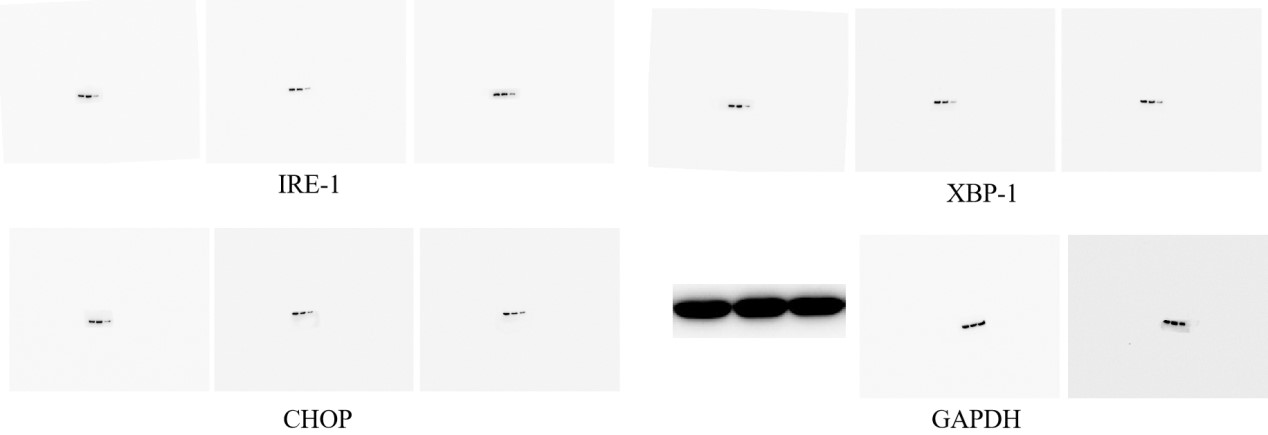


Fig 3E


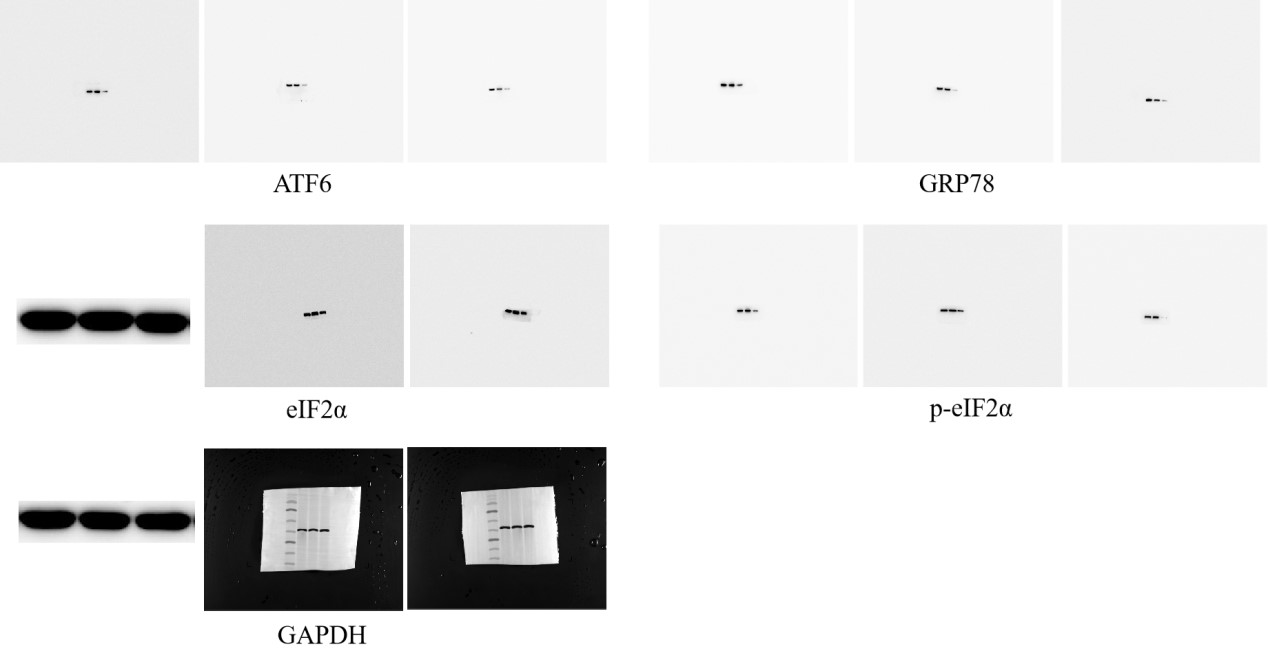


Fig 5F


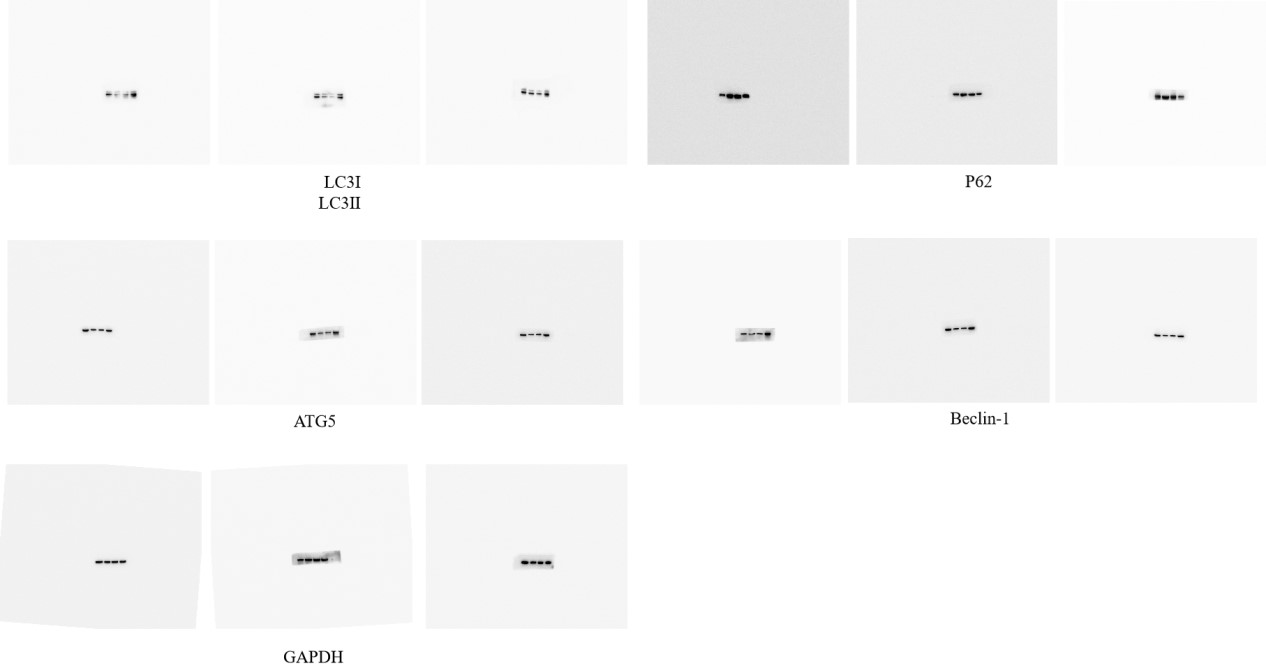


Fig 5G


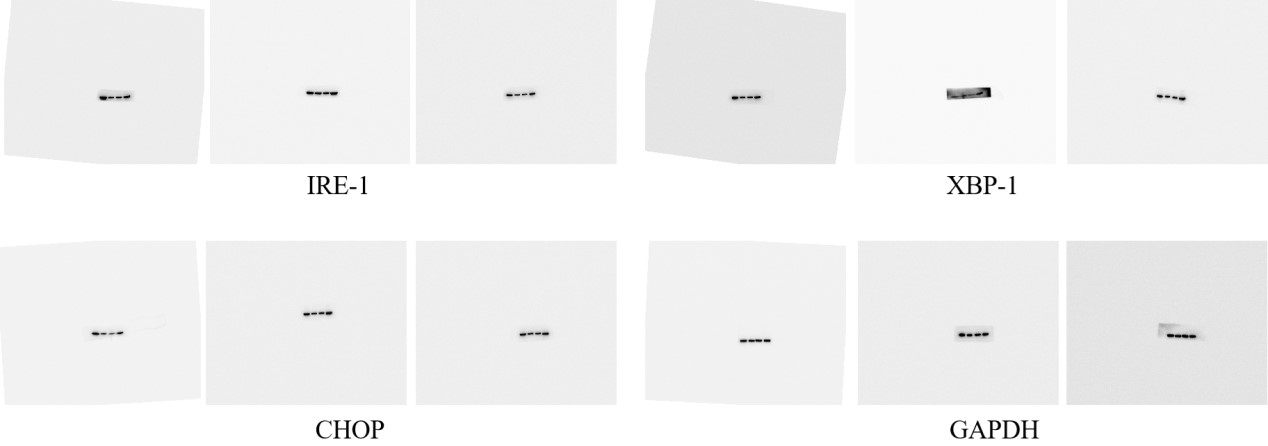


Fig 6G


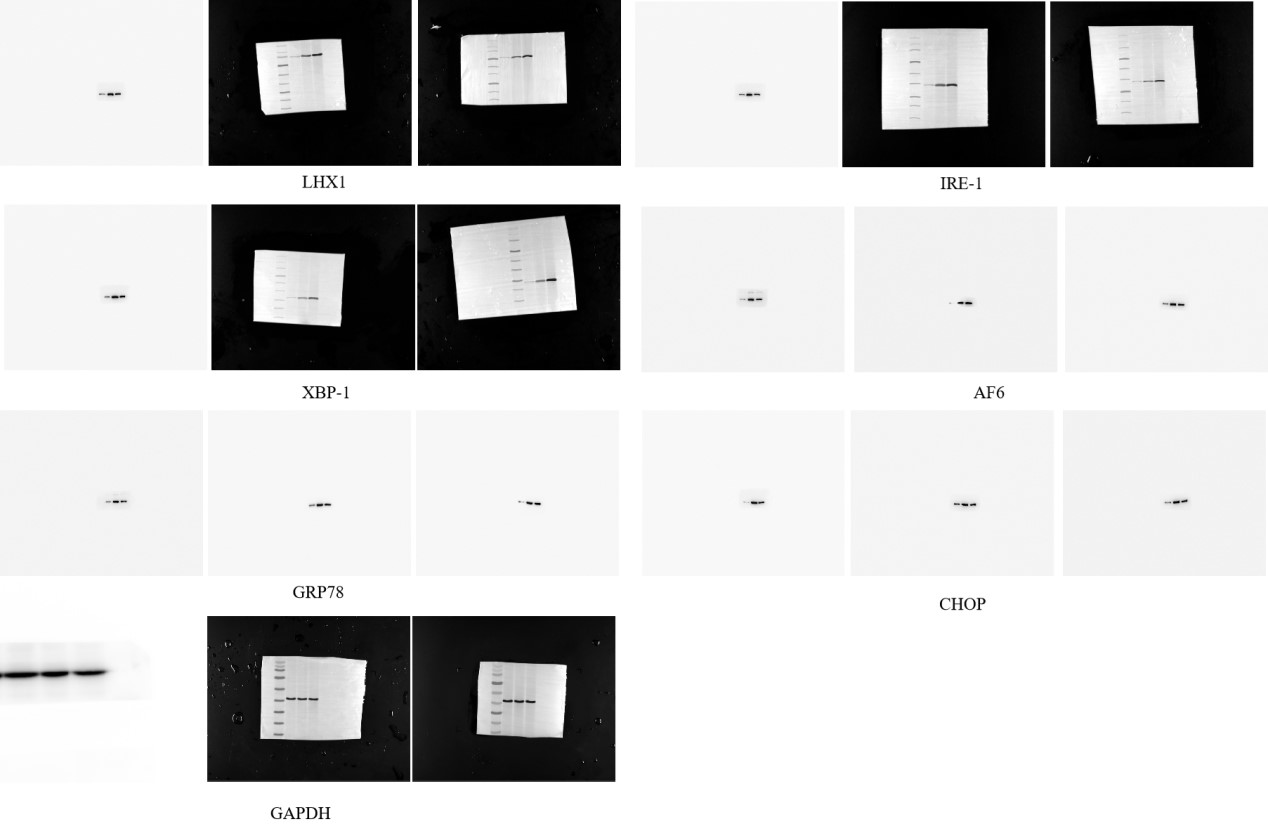

Supplement: Multimedia component 1 [file mmc1.docx]
