# Supplementary material for: LIM homeobox 1 (LHX1) induces endoplasmic reticulum stress and promotes preterm birth
Source: Heliyon. 2024 Jun 18;10(13):e32457. doi: 10.1016/j.heliyon.2024.e32457 (PMC467042; doi:10.1016/j.heliyon.2024.e32457)
Supplement: Multimedia component 2 [file mmc2.docx]

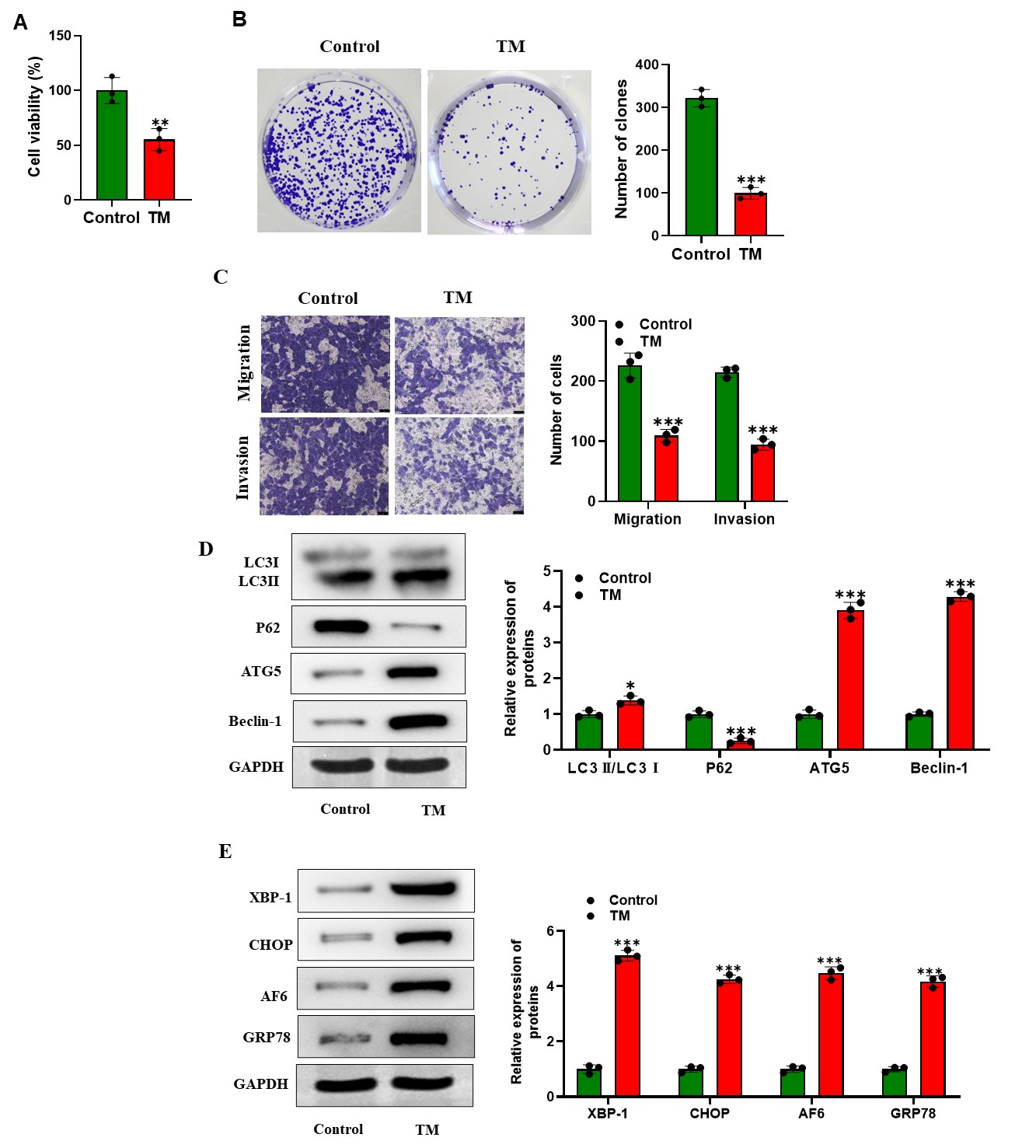


***Figure S1: Effects of TM on Trophoblast Cells.*** *(A-B) Assessment of cell viability and proliferation in HTR8/Svneo cells treated with TM using CCK-8 and colony formation assays, respectively (n=3 per group), highlighting a significant reduction in both cell viability and proliferation in response to TM treatment compared to the control group. (C) Transwell assays were employed to evaluate the migratory and invasive capabilities of HTR8/Svneo cells after TM treatment (n=3 per group). (D) Western blot analysis was conducted to detect protein levels of LC3, P62, ATG5, and Beclin-1 in HTR8/Svneo cells post TM treatment (n=3 per group), revealing a TM-induced elevation of autophagy through altered expression of key autophagic markers.* *(F) The protein levels of ERS markers, including XBP-1, CHOP, AF6, and GRP78, in HTR8/Svneo cells after TM treatment were assessed by western blot (n=3 per group), highlights the observed upregulation of these markers, underscoring the induction of ERS upon TM treatment.*P < 0.05, **P < 0.01, ***P < 0.001 vs control.*
